# Particle Localization Using Local Gradients and Its Application to Nanometer Stabilization of a Microscope

**DOI:** 10.1021/acsnano.2c09787

**Published:** 2022-11-16

**Authors:** Anatolii V. Kashchuk, Oleksandr Perederiy, Chiara Caldini, Lucia Gardini, Francesco Saverio Pavone, Anatoliy M. Negriyko, Marco Capitanio

**Affiliations:** † Department of Physics and Astronomy, University of Florence, Via Sansone 1, Sesto Fiorentino, 50019, Italy; ‡ LENS, European Laboratory for Non-Linear Spectroscopy, Via Nello Carrara 1, Sesto Fiorentino, 50019, Italy; ¶ Institute of Physics NASU, 46 Nauki Avenue, Kyiv, 03680Ukraine; § National Institute of Optics, National Research Council, Largo Fermi 6, 50125, Florence, Italy

**Keywords:** particle tracking, microscope stabilization, 3D localization, radial symmetry, local gradients, fluorescence microscopy

## Abstract

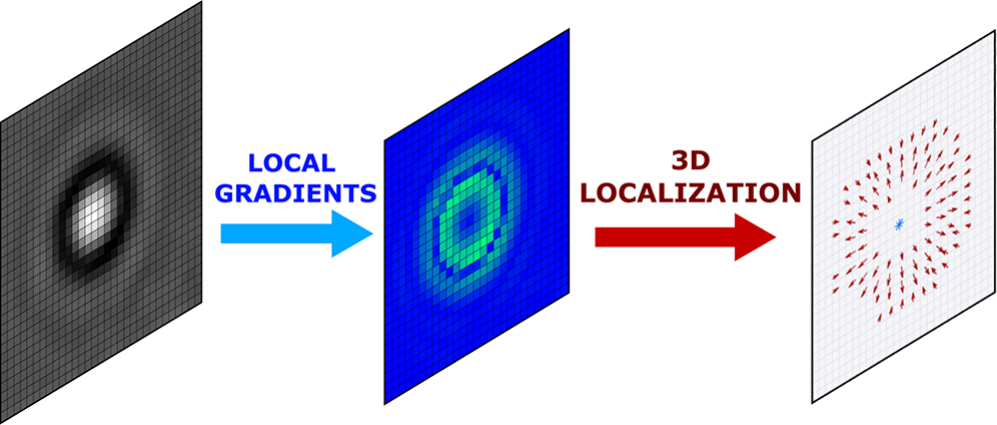

Particle localization plays a fundamental role in advanced biological techniques such as single-molecule tracking, superresolution microscopy, and manipulation by optical and magnetic tweezers. Such techniques require fast and accurate particle localization algorithms as well as nanometer-scale stability of the microscope. Here, we present a universal method for three-dimensional localization of single labeled and unlabeled particles based on local gradient calculation of particle images. The method outperforms state-of-the-art localization techniques in high-noise conditions, and it is capable of 3D nanometer accuracy localization of nano- and microparticles with sub-millisecond calculation time. By localizing a fixed particle as fiducial mark and running a feedback loop, we demonstrate its applicability for active drift correction in sensitive nanomechanical measurements such as optical trapping and superresolution imaging. A multiplatform open software package comprising a set of tools for local gradient calculation in brightfield, darkfield, and fluorescence microscopy is shared for ready use by the scientific community.

Localization of micro- and nanoparticles has a broad applicability and plays a significant role in different physical and biological methods. Some examples include position and force measurement in optical tweezers,^1,2^ investigation of motility of swimming microorganisms,^3^ tracking of objects in microfluidic devices,^4^ study of dynamics of proteins and vesicles in cells,^5,6^ and superresolution fluorescence microscopy.^7^ Some advanced imaging techniques, such as single-molecule localization microscopy,^8^ heavily rely on accurate subpixel localization of fluorophores with numerous tools available for superresolution image reconstruction.^9−11^

Another class of applications in which a position of particles needs to be determined quickly and precisely is the active mechanical stabilization in optical microscopes.^12^ In experiments that require nanometer or subnanometer stability a feedback system is crucial. For such setups, the thermal drift of the viewing plane due to heating/cooling of the optical elements creates significant issues. This occurs, for example, in nanomechanical measurements performed with optical and magnetic tweezers or atomic force microscopes.^13^ If the imaging system is combined with optical tweezers, thermal drifts become even more severe due to the presence of a high-power laser beam. A similar problem arises in single-molecule localization microscopy (SMLM) as methods like STORM (stochastic optical reconstruction microscopy) and PALM (photoactivated localization microscopy)^14^ require high power of the excitation beam and long acquisition times and, therefore, will greatly suffer from an imaging plane drift. While there are algorithmic solutions to correct for this displacement after image acquisition, it is not always feasible, as the drift might be too large to be compensated in postprocessing. Moreover, this approach is not applicable to nanomechanical measurements. An active stabilization system that controls the position of the objective or sample chamber can compensate for the drift in the recorded data in the first place. Most commonly such systems utilize a bead or fluorescent marker attached to a coverslip as a reference for correction and require the position of the particle to be measured in three dimensions.^15,16^

The most common way to localize a single particle is to apply a threshold to select the brightest pixels in the image followed by calculation of an intensity-weighted centroid. Despite being very fast, this method shows poor performance and has several practical issues.^17^ The aforementioned tracking algorithms in superresolution fluorescence microscopy were developed for a specific task of image reconstruction for fluorescent probes and are unsuitable or poorly applicable in other cases. Several gradient-based methods calculate the difference between adjacent pixels to find direction and magnitude of the intensity gradients in the image. Given that in most cases particles are imaged as objects with radial symmetry, their location can be determined as an intersection of gradient lines.^18,19^ This approach is experimentally convenient, as it is invariant to illumination variation and independent of background level. Also gradient methods were extended to 3D detection of fluorescent particles.^20^ In astigmatism-based microscopy a gradient fitting algorithm was employed to provide 3D localization of fluorophores.^21^ Overall, gradient algorithms provide accurate computationally efficient methods for fluorescent particle tracking and thus are widely used for fast localization in fluorescent microscopy. Another interesting approach was proposed in the DeepTrack software,^22^ which utilizes recent advances in convolutional neural networks to localize particles of different types, shapes, and sizes. However, as with all artificial neural networks, it requires training data to operate and its performance heavily relies on the size and quality of the data set provided.

Here, we present methods for particle localization and microscope stabilization based on the calculation of local gradients of the image intensity. In general, the local gradient algorithm (LoG) can be useful in different applications in image processing that require calculation of gradients. However, for the purpose of this paper, we will focus solely on the use of local gradients in particle localization tasks. We propose a set of tools for 3D localization of both fluorescent and unlabeled particles. The software was primarily developed for active stabilization systems in sensitive biological experiments and, therefore, includes parameters that can be easily adapted to specific conditions, executes in a short time frame to allow high processing rates, and provides accurate results at low signal-to-noise ratios. However, the LoG algorithms presented here have a much more broad applicability. We test and demonstrate usability of the LoG algorithm in XYZ-localization of particles in brightfield and darkfield imaging and fluorescent particles in astigmatism-based microscopy.^23^

To make the software more suitable for immediate incorporation, the LoG tools are available on the platforms that are commonly used for data capture and analysis: Matlab, LabVIEW, and Python. The software can be obtained from refs (24) and (25).

## Results and Discussion

**Figure 1:**
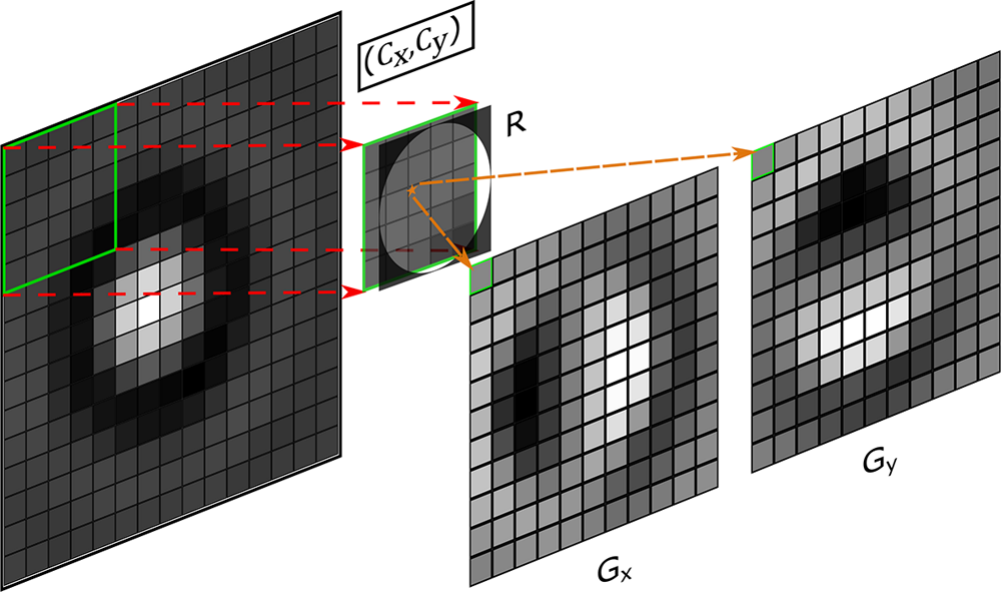
Visualization of the local gradient algorithm. For an *m* × *n* image (here 15 × 15) a centroid of a sliding window size  (here 5 × 5 with *r* = 2.5) is calculated. Each pixel of gradients *G*_*x*_ and *G*_*y*_ is determined as the *x* (*C*_*x*_) and *y* (*C*_*y*_) coordinates of the centroid, correspondingly, which is calculated relative to the center of the window. The resulting matrices *G*_*x*_ and *G*_*y*_ have the size (*m* – *K*) × (*n* – *K*). Negative gradient values for images *G*_*x*_, *G*_*y*_ are represented as darker pixels and positive as whiter pixels. An orange star depicts the centroid. *R* is a circular mask *K* × *K* of radius *r*.

We define a local gradient in a given point as the intensity-weighted centroid of all the pixels within a radius *r* from that point (see Figure 1). By calculating local gradients for each pixel we obtain horizontal and vertical gradient matrices (*G*_*x*_ and *G*_*y*_ in Figure 1) of the original image (see Supporting Information for more detailed derivation).

The use of gradients to localize radially symmetrical single particles has been shown to be a robust and effective method.^18,19^ Though such methods demonstrate high accuracy and stability to brightness variations, they are susceptible to noise and show poor performance at low signal-to-noise ratios. Also most of them lack flexibility due to employment of a fixed-size kernel for gradient calculation. In the proposed LoG algorithm the radius of the window *r* determines the number of pixels included in the calculation of local gradients. Therefore, by adjusting *r* one can enhance a calculated gradient for an object of a specific size. Moreover, this provides much better results for images with low SNR as more pixels will be averaged.

We show and thoroughly test several approaches based on LoG algorithms to determine the 3D position of particles in brightfield, darkfield and fluorescent microscopy.

### 3D Particle Localization in Brightfield Microscopy

**Figure 2:**
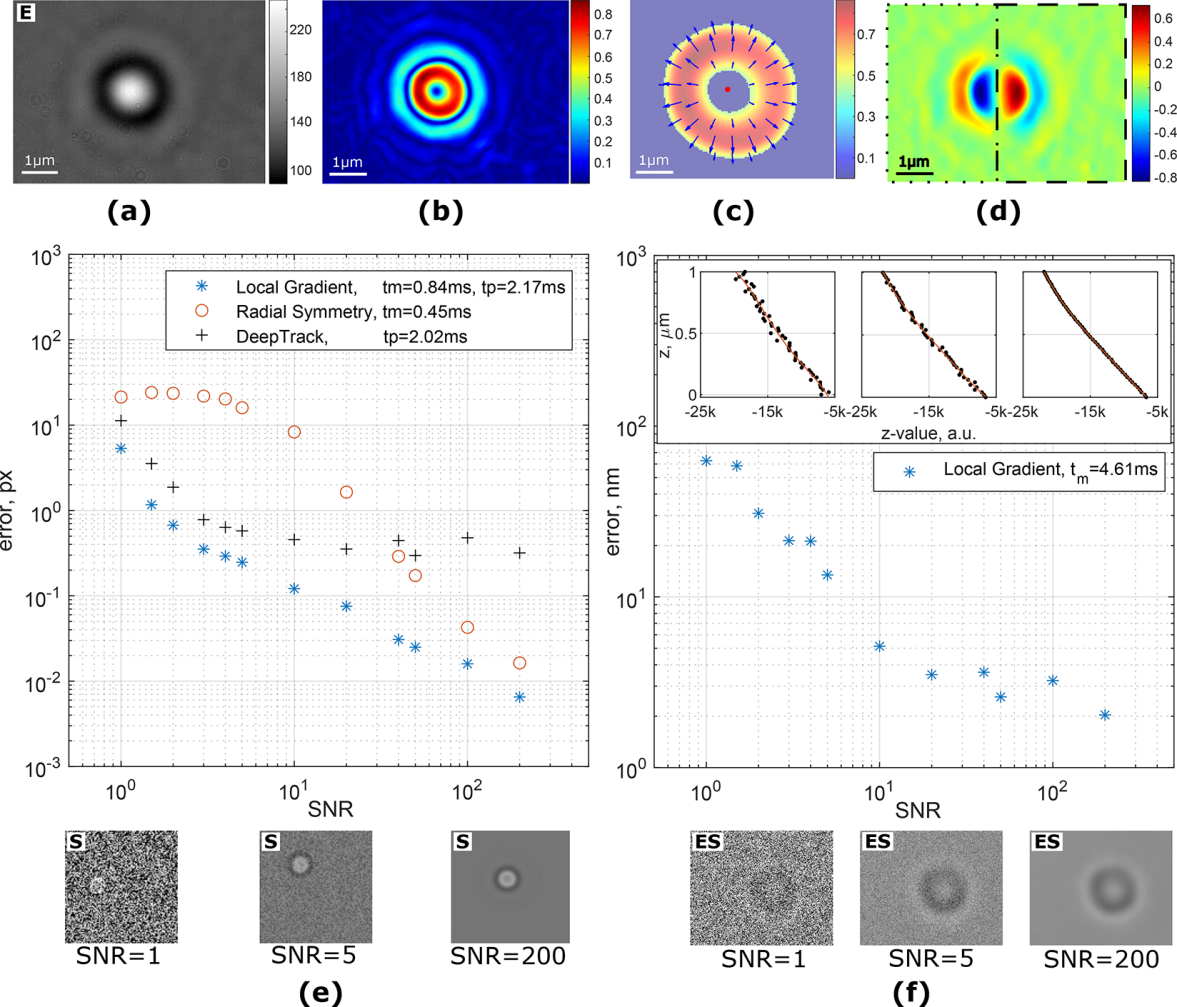
Localization of a particle using a local gradient algorithm (images a–d are not to scale for better visualization). (a) Input image of the 0.9 μm silica particle. (b) Magnitude of local gradients. (c) Magnitude of local gradients after thresholding. Arrows show the direction of gradients (from high to low). The position of the particle (red spot) is determined as a least-squares intersection of all gradient vectors. (d) *z*-Value calculation. Local gradient *G*_*x*_ is split into left (dotted) and right (dashed) parts. *z*-Value is determined as a difference between the sums of gradient values of “right” and “left” sides. (e) Accuracy and execution time performance of XY-localization algorithms and different SNRs. Each error point is an average error of 20 images. tm and tp are average execution times in Matlab and Python, correspondingly. Example of the test images (scaled from 0 to 1) is shown under the plot. (f) Accuracy and execution time of the LoG algorithm for *z*-localization. Inset shows examples of calibration curves for SNR = [2, 5, 200].

Figure 2a–c depicts the localization of a 0.9 μm silica particle from its brightfield image (Figure 2a).

The gradient matrices *G*_*x*_ and *G*_*y*_ calculated from the LoG algorithm using eqs S3 and S4 form a vector field (as depicted by blue arrows in Figure 2c) that contains a gradient vector for each pixel. The center of a radially symmetrical particle can be determined as an intersection of all gradient lines. However, the presence of the noise or uneven illumination in the background of the image will create gradient vectors with random or incorrect orientation that may disrupt the estimation of the center. To limit the influence of such artifacts on the localization, the magnitude (Euclidean norm) of gradient vectors is used to exclude low-magnitude values (Figure 2b,c). The calculation of an intersection point of gradient lines implies solving a system of linear equations. In practice such system is overdetermined and inconsistent, as it is very unlikely that all the gradient lines will intersect at a single point. Therefore, the center of the particle is calculated using the method of least-squares applied to gradient lines with the highest magnitude of gradient vectors (see Supporting Information).

To investigate the performance of the LoG algorithm for *xy*-localization, we run a set of tests and compare the results to other available methods: radial symmetry algorithm^18^ and DeepTrack software.^22^ The first method uses a similar approach in the calculation of the center of the particle—least-squares intersection of gradient lines—but significantly differs in the gradient calculation. In the radial symmetry algorithm the gradients are calculated between adjacent pixels only, which leads to an increase in the detection error due to noise amplification for images with low signal to noise ratio (SNR). The second software package, DeepTrack, provides tools for training and validation of a convolutional neural network (CNN) for particle tracking. To compare our results with DeepTrack, we have trained a network using built-in tools feeding 30 000 images of simulated particles randomly positioned and with added noise (see Methods). It should be noted that the performance of the DeepTrack network, as in any CNN, strongly depends on the type and size of the training data. Thus, the test results for a given network will serve as a reference of what is available out of the box and not necessarily reflect the full capability of the software. As DeepTrack is based on Python, and the radial symmetry algorithm is written in Matlab, we compare each of the software packages with a corresponding implementation of the LoG algorithm.

The test results are shown in Figure 2e. The LoG algorithm demonstrates an excellent noise stability and outperforms both radial symmetry and DeepTrack in most cases. The execution time of all methods is comparable for a given platform. A precalculation of the Fourier transform of matrices in eq S5 saves 0.30 ms (∼24%), which may speed up the execution time when using the same parameters for multiple images.

The axial position of the particle in brightfield microscopy is a more complex task. Using local gradients we have devised a simple yet fast and effective approach to measure the *z*-position. The basic concept is shown in Figure 2d. A horizontal local gradient image *G*_*x*_ is split into two parts relative to the center of the particle. The difference between the sum of all gradient values on the “right” and “left” sides provides an excellent metric for a calibration curve or look-up table.

A similar metric can be built for the vertical gradient *G*_*y*_, and an average of both horizontal and vertical gradients constitutes a *z*-position value. The test results of the noise performance shown in Figure 2f were performed using real images of a single particle with artificially added noise. A set of 101 images is taken at different heights with step size of 10 nm. In order to differentiate input data, each odd image was taken to create a calibration curve, while each even image is used for the test (see Methods).

As there are no “pixels” in the axial direction, we conclude the test results based on the nanometer position of the objective piezoscanner. With such a simple approach we are capable of achieving an error as low as ≈2 nm in the case of high SNR images.

### 3D Particle Localization in Fluorescence and Darkfield Microscopy

**Figure 3:**
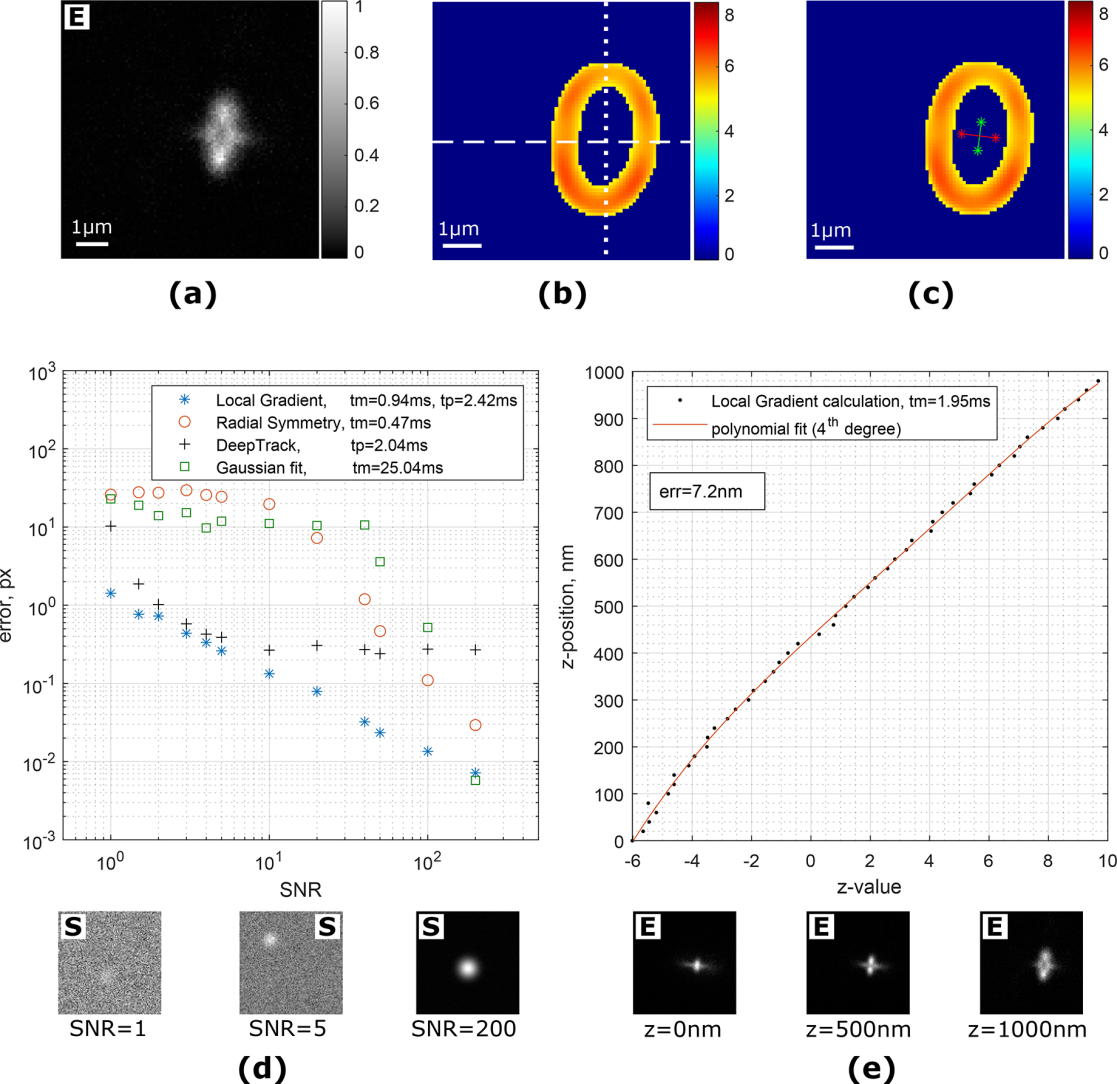
(a) Image of a single fluorescent particle (polystyrene, 0.51 μm) attached to a coverslip. Astigmatism is introduced by a cylindrical lens, and the imaging plane is ≈500 nm above the surface. (b) Magnitude of local gradients. Dashed and dotted lines are showing the top/bottom and left/right split of the local gradient images for *z*-value estimation, correspondingly. (c) Two axes (green and red lines) are built from the centers of split gradient lines. (d) Comparison of algorithms for localization of a simulated Gaussian-like particle at different noise levels. tm and tp are average execution times in Matlab and Python, correspondingly. Examples of the test images are shown under the plot. (e) *z*-Value calibration curve in astigmatism-based microscopy. The average error for predicting a *z*-position of the particle is 7.2 nm. Examples of the test images are shown under the plot. “S” and “E” denote simulated and experimental images.

Determination of the position of fluorescent particles and markers in *x* and *y* is a very similar task to the localization of particles in brightfield microscopy. Hence, the same methods can be applied. The difference between the two is that fluorescent images, in particular when a sample is at a single-molecule concentration, are more noisy due to a typically low signal from fluorophores. Figure 3d demonstrates a comparison of accuracy of local gradient method, radial symmetry, DeepTrack, and Gaussian fitting for generated Gaussian-like fluorescent particles (see Methods).

Measurement of the axial position in the case of fluorescent particles is much more challenging. Unlike the image of micron-sized beads in brightfield microscopy, the point spread function (PSF) of fluorescent particles with subdiffraction dimensions does not vary significantly in shape at different heights near the objective focal plane. This causes the particles below and above the imaging plane to look the same and makes them very hard to properly localize. The solution to this issue is to defocus the image^26,27^ or introduce some sort of aberrations into the imaging system to break the axial symmetry of the PSF.^28,29^ In the latter case an engineered PSF is used to induce an axially dependent feature. One of the methods to provide sensitivity in axial position for fluorescent probes is to use a cylindrical lens in the optical path.^23^ The PSF remains roughly round only when the particle is in the imaging plane and becomes elliptical when outside with a major axis flip of 90 deg when passing through the imaging plane (see Figure 3e). It has been shown that in such a system the *z*-position is proportional to the ellipticity of the PSF. The LoG algorithms provide an opportunity to utilize their features to create a metric for fast and efficient axial localization. A local gradient image of the fluorescent particle with introduced astigmatism can be easily made to resemble an ellipse by adjusting the window size *r* (Figure 3b).

The calculation procedure of the axial metric is shown in Figure 3b,c. The image is thresholded by the magnitude of the gradient. Once the *xy*-position has been found, the image is split into top/bottom or left/right sides relative to the center of the particle. For each part a least-squares intersection of gradient lines is calculated, resulting in four points, which form two axes. The length of the major axes is taken as a *z*-value (see Supporting Information for a detailed description).

The results of the noise tests for *xy*-localization (Figure 3d) show that, again, the LoG algorithm outperforms both DeepTrack and Radial Symmetry at all tested SNRs while keeping the execution time comparable to other methods. For *z*-position measurements the proposed *z*-value metric provides a linear response within the tested 1000 nm displacement with an average execution time of 1.96 ms per image. The averaged error in position determination is *z*_err_ = 7.2 nm.

The algorithm used for localization of fluorescent particles using astigmatism can be used also for 3D particle localization using darkfield microscopy. We have tested this by localizing 60 nm gold nanoparticles in a commercial darkfield microscope (see Supporting Information).

**Figure 4:**
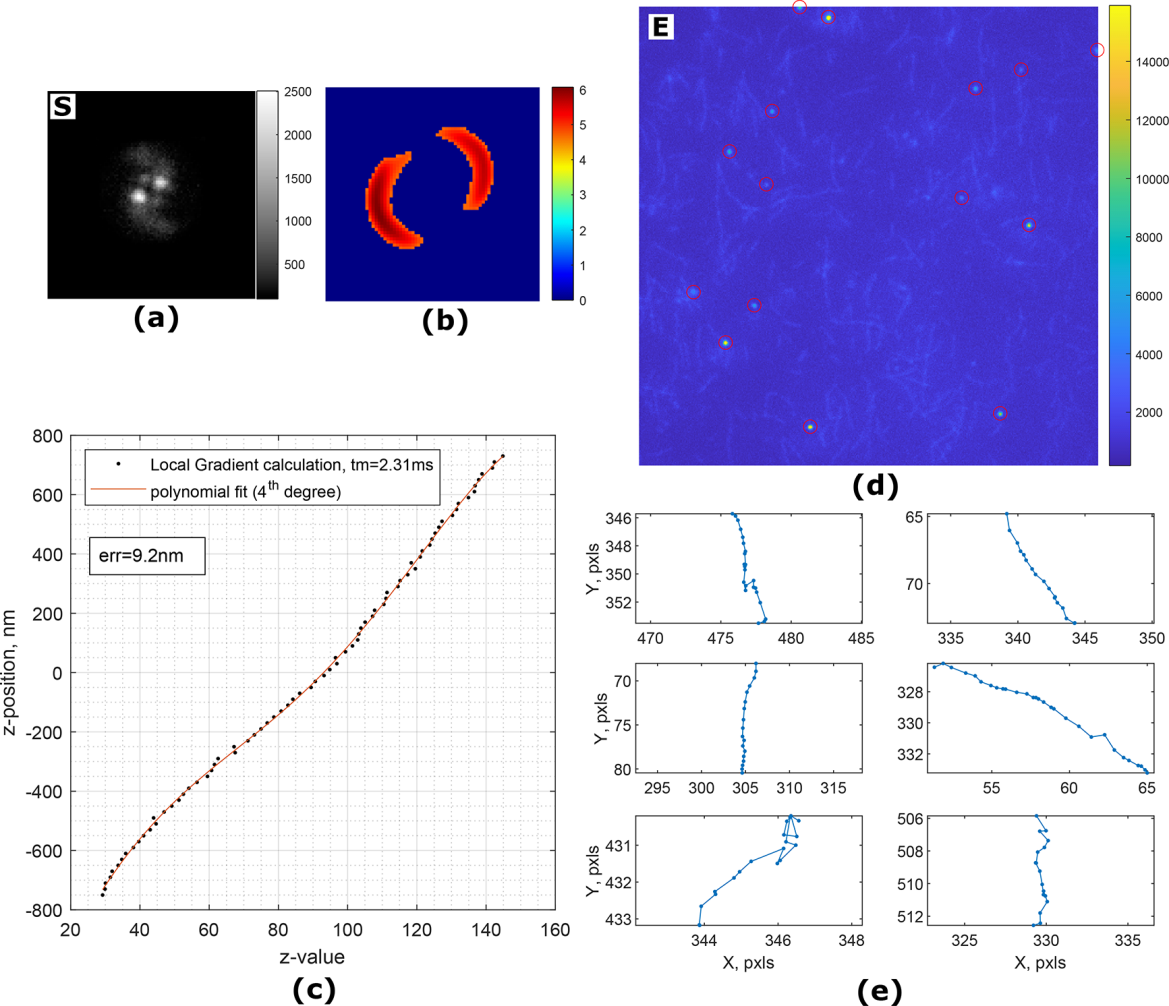
(a) Simulated image of a particle with double-helix PSF. “S” and “E” denote simulated and experimental images. (b) Corresponding magnitude of calculated local gradients with applied threshold (*R* = 15, *thr* = *max*/1.3). (c) Axial calibration curve. tm corresponds to average execution time per image in the Matlab environment. (d) Image of single myosin-5B molecules labeled with quantum dots. Red circles depict localized molecules with the local gradient algorithm. (e) Examples of reconstructed trajectories of moving myosin-5B.

Another commonly used method for 3D localization utilizes a double-helix (DH) PSF.^30−32^ The DH-PSF appears as a 3D double-helix that rotates along the optical axis. For a given *z*-slice a double-helix is represented by two lobes that form an angled line (Figure 4a). The orientation of the line depends on the axial position of the emitter and, thus, provides *z*-localization. As with an astigmatism-based microscopy, we can use the local gradient algorithms to perform *z*-localization of the fluorescent particle. Choosing large window size *R* and high threshold allows conversion of two lobes into an ellipse-like structure. The angle between the lobes can be easily calculated using central moments of the thresholded gradient magnitude image (Figure 4b). To test our algorithm, we use a generated data set for DH-PSF microscopy.^33,34^ A stack of images in the 3D-double-helix microscopy were simulated in 10 nm steps in the range [−750 nm; 750 nm]. The calibration curve is shown in Figure 4c.

### Multiparticle Tracking

Local gradient algorithms can be used for tracking of multiple particles. In this case the magnitude of local gradients is used to isolate individual particles, and a density-based spatial clustering algorithm^35^ is applied to select the gradients that correspond to a single particle. Figure 4d demonstrates localization of several single myosin-5B molecules labeled with quantum dots.^36^ Images in the data set were recorded under total internal reflection fluorescence (TIRF) microscopy. Examples of reconstructed trajectories of myosin-5B molecules moving along the immobilized actin filaments are shown in Figure 4e. The developed algorithm shows good results for localization and tracking of particles with low density and can be used to track multiple particles in an online regime. However, overlapped or closely situated particles will be most likely detected as a single particle due to an overlap of their gradient lines. Moreover, single-particle tracking of rapidly moving objects could pose more severe requirements for the number of photons detected per frame and the temporal resolution to obtain high localization precision and avoid blurring or distortion of the PSF. Assuming that the SNR is limited by the photon count, as is usually the case in single-molecule tracking based on fluorescence (Poisson statistics), the SNR is proportional to the square root of the number of photons detected per camera frame. Therefore, the localization error plotted in Figure 3 can be directly related to the number of detect photons, which can serve as guidance to adjust the integration time and sensitivity of the camera.

### Nanometer Stabilization in Brightfield Microscopy for Nanomechanical Measurements

**Figure 5:**
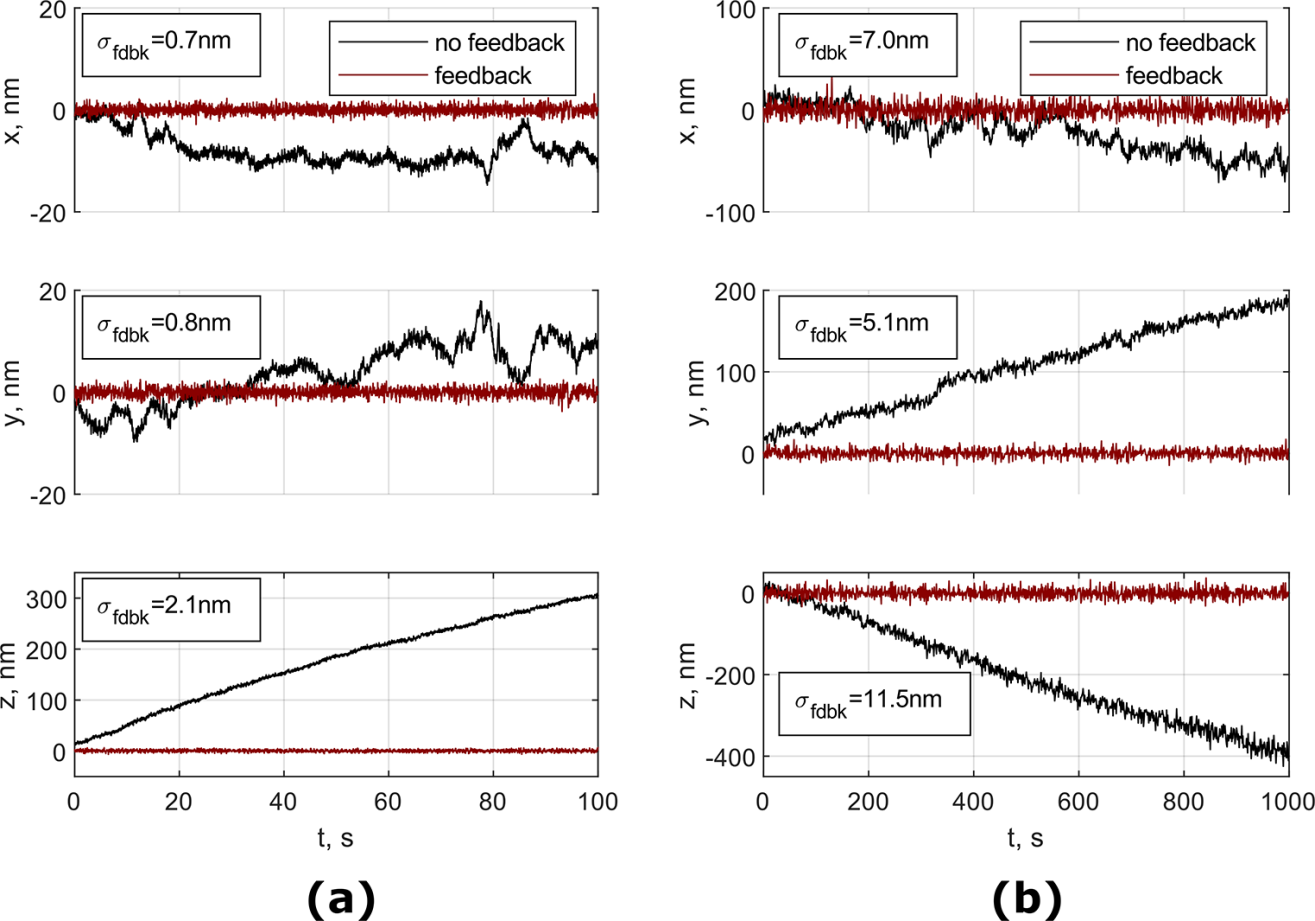
3D tracking of a spherical silica particle in brightfield microscopy (a) and a fluorescent polystyrene particle in astigmatism microscopy (b) that were attached to the coverslip with feedback system on and off. Inset indicates the standard deviation of the signal with feedback on.

Finally, the *xyz* algorithms for brightfield images described above were applied for a feedback system to stabilize the drift of the imaging plane and reduce the impact of mechanical noise. The experimental setup was an ultrafast force-clamp spectroscopy system,^37^ which is used to study protein interactions under a constant load. In these experiments, a “dumbbell” structure consisting of an actin,^38−40^ microtubule,^41^ or DNA filament^42^ strained between two microbeads is held by optical tweezers. The actin filament is brought into the vicinity of a stationary microbead that is attached to the coverslip and covered with proteins of interest. As the interaction area between the filament and the protein is often on the order of nanometers, a mechanical stabilization is critical for protein attachment/detachment to be observed and measured. Moreover, measurement of nanometer-sized conformational changes of proteins critically relies on the mechanical stability of the system. Therefore, in these experiments the stationary microbead is used as a fiducial mark for microscope stabilization.^43^ Here, we demonstrate the use of local gradients for the mechanical feedback system in stabilization of the viewing plane (see Methods). Figure 5a shows tracking of a single particle attached to the coverslip with and without feedback.

Our feedback system demonstrates the ability to suppress mechanical noise and thermal drift to subnanometer levels in *xy*-localization and to 2 nm in axial positioning. As can be seen from the plots, the displacement of the viewing plane with no feedback creates a sufficient movement, on the scale of tens to hundreds of nanometers, which makes protein interaction measurements in force-clamp setups impossible to do.

### Nanometer Stabilization in Superresolution Microscopy

A similar approach was used to test the performance of the feedback system using the LoG algorithm on fluorescent particles. This test was performed on an inverted fluorescence microscope for superresolution microscopy (see Methods) on a sample made of commercial 500 nm fluorescent polystyrene beads (Exc/Em 480/520) attached to a glass coverslip (see Methods). A single particle was tracked in 3D for 1000 s (a representative time for STORM image acquisition). The calibration curve for the *z*-value was recorded on the same bead before the acquisition. The results for both feedback-controlled and free-running cases are shown in Figure 5b. Again, we were able to demonstrate a stable positioning of the sample with a standard deviation of the position in the range of 5–7 nm for *x*–*y*-localization and 11.5 nm for *z*-localization.

**Figure 6:**
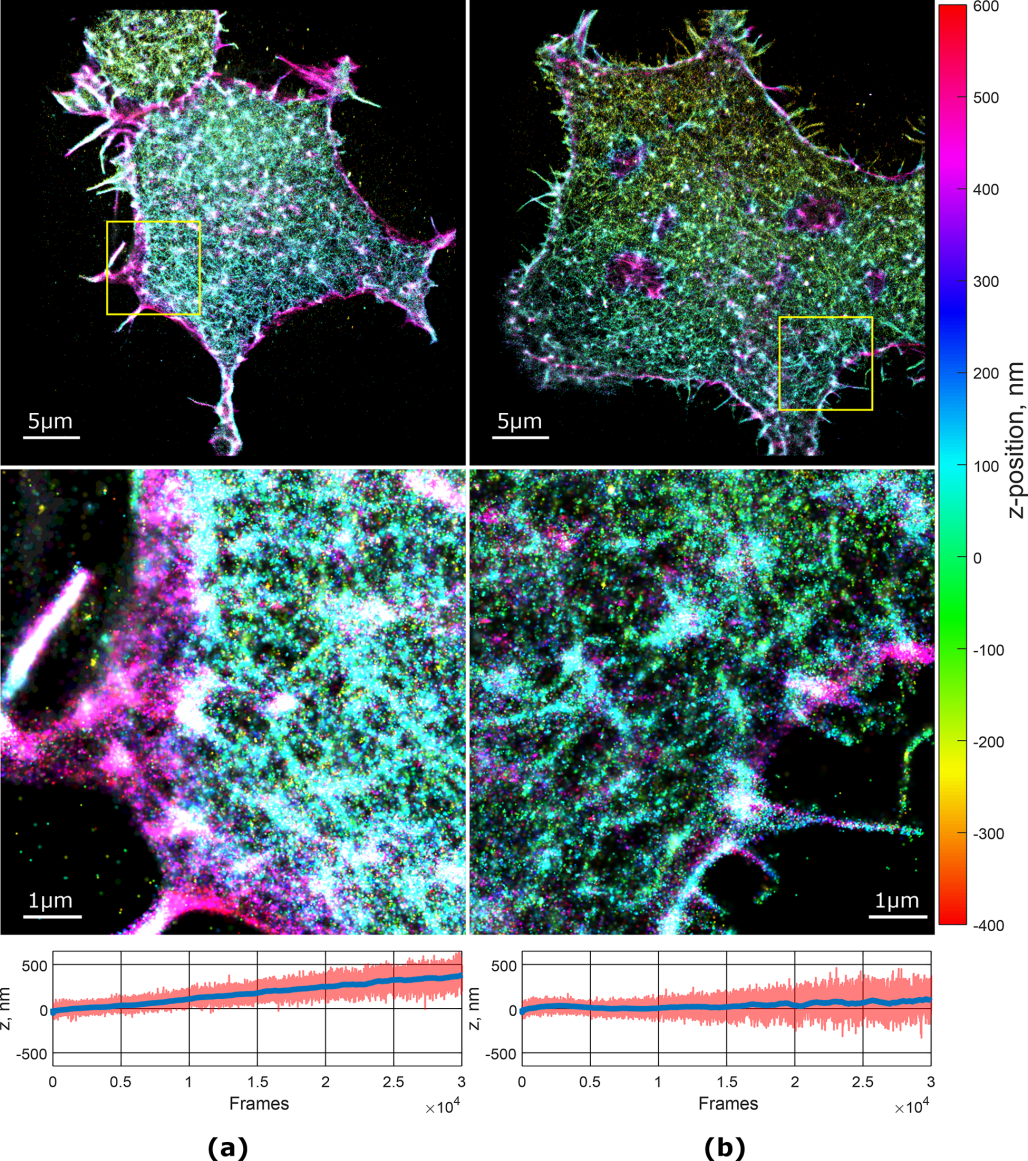
Reconstructed 3D STORM images of cells without feedback correction (left) and with feedback (right). Plots under the images show the change in the average *z*-position (red line) of all detected fluorophores (blue line represents moving average of 1000 points).

Next, we applied the feedback system to record a 3D-STORM image of the actin cytoskeleton of a mammalian cell using a fluorescent bead as a fiducial marker. A 3D-STORM image of the actin cytoskeleton of a different cell from the same sample was recorded without the feedback for comparison (Figure 6a).

Additionally, we estimate the drift in the *z*-axis by calculating the average position of all detected fluorophores for each frame. The actin cytoskeleton has an uneven distribution within the cell volume, with a dense branched cortex spanning about 200–300 nm from the cell membrane and a less dense network protruding toward the cell nucleus. If the viewing plane is drifting axially, the average *z*-position of all fluorophores in a frame will drift as well. The axial drift is clearly visible in the images acquired in the absence of feedback, contrary to what is observed with the feedback (plots in Figure 6). As a consequence, the image acquired with the feedback (Figure 6 on the right) appears sharper and shows more fine details compared to the one without feedback.

## Conclusions

The localization methods based on gradient calculations offer several advantages. Low dependence of the gradients on the background intensity level makes them suitable for particle detection in changing conditions or uneven illumination. Furthermore, these methods are applicable to *xy*-detection of particles that are only partially present in the field of view of the camera, as even a small number of gradient lines will point toward the center. This property can be useful in the case of a limited field of view or obstruction of the particle by other objects. Noniterative algorithms provide fast execution with accuracy comparable to fitting algorithms. Not surprisingly, such algorithms are widely used in fluorescence microscopy for subpixel localization of fluorophores. Given a typical size of an imaged single fluorophore of a few pixels, gradient-based algorithms are usually a good choice when short execution time is required. Consequently, most of the existing gradient methods are optimized for fluorescence microscopy. A radial symmetry algorithm^18^ uses a pixel-to-pixel difference to calculate gradients, an approach that works well for high-SNR images that consist of few pixels but fail in low-signal conditions and for larger particles. An optimized algorithm^21^ uses a fixed Sobel-like 4 × 4 kernel to calculate gradients which also allows axial localization. The introduced local gradients are based on a customizable kernel. The mentioned difference in the gradient calculation appears as a small improvement of the previously developed methods, but this improvement has huge implications on the localization. The variability of the window size *r* allows selective enhancement of the objects of particular size (see Supporting Information). This allows the optimization of the window size to reduce image noise or select a particle of specific size in strongly polydisperse samples. This feature also helps in removing small dust spots from the brightfield images. More importantly, by adjusting the window size we can enhance the image features that are important for axial localization of the particles. This is especially crucial for various fluorescence techniques with a modified PSF approach such as double-helix and astigmatism-based microscopy. We have developed and tested several methods for 3D particle localization in brightfield, darkfield (Supporting Information), and fluorescence microscopy utilizing local gradients.

In comparison with other methods, LoG algorithms are more noise resistant and show better accuracy for all tested cases. While DeepTrack can be trained to track much broader classes of objects, LoG software is more flexible and provides better control over the tracking process for radially symmetrical particles. For mechanical stabilization systems the execution time is an important parameter for the localization software to reach a sufficient feedback frame rate. In LoG methods the execution time mostly depends on the input image size, as the most time-consuming steps are convolutions represented as a series of forward and inverse Fourier transforms. It should be noted that the speed of FFT algorithms used for Fourier transform calculations has a complex dependence on the image size and can be further optimized by cutting/expanding the input image to the appropriate size.

For accurate determination of the axial position a calibration curve or look-up table should be created for each particle of interest, as small variations in size may lead to much higher errors than those obtained in the tests. The axial calibration variation section in the Supporting Information demonstrates the variation in the calibration curves of different spherical gold nanoparticles recorded simultaneously in a darkfield illumination. It is apparent that even in similar conditions the measured axial position has a significant variation between the particles, which implies that each particle should have individual calibration. However, the main difference between the calibration curves is in horizontal bias, while the shape and slope of the curves are preserved. In most cases a mechanical feedback system does not require an absolute value of the axial position but locks on a specific position. Given the similarity in the calibration curves, it is possible to apply a prerecorded calibration to similar particles. The main requirement for the metric in this case is a monotonic output across the possible axial ranges, which our algorithms immaculately fulfill even when the viewing plane is crossing the center of the particle.

The LoG algorithms were primarily developed for feedback systems that require fast and precise determination of the 3D position of the particle. In microscope stabilization tasks there are various approaches to fiducial mark localization. For fluorescent particles a 3D position can be estimated from the Gaussian fit.^15^ In brightfield microscopy correlation methods can be used to estimate a 3D drift of the sample.^16^ While these methods demonstrate good performance for the specific task, they are not universal. The LoG algorithms provide tools for a wide range of microscopy techniques covering brightfield, darkfield, and fluorescence imaging. We have demonstrated an efficient subnanometer stabilization of a microscope stage that is used for force spectroscopy of proteins. It is clear that a free-running system will not allow any protein interaction measurements due to a significant drift which will quickly move the proteins out of interaction region. A similar problem exists in superresolution microscopy in which a stack of images is recorded (such as PALM/STORM). In many cases the movement of the viewing plane can be corrected in postprocessing by drift-correcting algorithms. These algorithms are especially effective in *xy*-axes but work only in a limited range of axial drift as the SNR of out-of-focus chromophores quickly decreases, making them undetectable. We have shown that a feedback system based on local gradient algorithm shows significant improvement in *z*-axis stability and improves the overall image quality.

## Methods

All calculations were performed on Lenovo ThinkPad T15g Gen 1 Core i7-10850H vPro, Windows 10 Pro 64, 32 GB (2933 MHz).

### XYZ Detection Accuracy Tests

Images of particles for *xy*-performance tests in brightfield were obtained from a radial profile of a 3 μm silica bead (Bangs Laboratories, SS05001, M.D. 3.17 μm) fixed on the coverslip. A smoothing spline is applied to reduce a pixelation in the image. A set of 20 images (100 × 100 pxls) was generated for each SNR (12 SNR levels) with randomly positioned particles. SNR is defined as a ratio of the maximum signal to the standard deviation of the noise. The same set of images is used for all the software packages to test. The error is defined as a distance between the predicted and true location of the particle. Local gradient parameters used to track the particles are *r* = 10 and threshold cutoff 1/1.4 of maximum gradient intensity. The execution time was estimated by performing 200 runs of 240 generated images (48 000 runs in total). To equalize a cross-platform productivity, Tensorflow was forced to use a single core.

Axial positioning is tested on set of images (222 × 276 pxls) of 1 μm silica particle (Bangs Laboratories, SS0400, M.D. 1.05 μm) attached to the coverslip. The image of the particle is recorded at different heights with 10 nm steps using an objective scanner. The calibration is created by using every odd image (20 nm step, 51 images), and the tests are performed on each even image (20 nm step, 50 images). A random noise was added at specified SNR (12 levels). Local gradient parameters used to track the particles are *r* = 35 and threshold cutoff 1/1.5 of maximum gradient intensity.

### XYZ Detection for Fluorescent Particles

Images for the *x*–*y* positioning tests (100 × 100 pxls) were generated from high-resolution images (10000 × 10000 pxls) of a randomly distributed 2D Gaussian function. A set of 20 images is generated for each SNR (12 SNR levels) with randomly positioned particles. Local gradient parameters used to track the particles are *r* = 12 and cutoff threshold 1/1.3 of maximum gradient intensity. The execution time was estimated by performing 200 runs of 240 generated images (48 000 runs in total). Tensorflow was forced to use a single core.

Axial positioning is tested on a set of images of 500 nm fluorescent beads (100 × 100 pxls) attached to the coverslip. The image of the particle is recorded at different heights with 10 nm steps using an objective scanner. The calibration is created using every odd image (20 nm step, 51 images), and the tests are performed on each even image (20 nm step, 50 images). Local gradient parameters used to track the particles are *r* = 10 and cutoff threshold 1/2 of maximum gradient intensity.

Gaussian fitting was performed by nonlinear least-squares fitting to a 2D Gaussian function with a termination tolerance of 0.0001.

### Training of the DeepTrack CNN

The DeepTrack software^22^ was used to create a convolutional neural network for particle detection. Along with the amount, quality, and variety of the data used to train the neural network, the architecture of the neural network, its training parameters, and conditions are key factors in obtaining a good result. Here we build and train the network similarly to the examples and tutorials shipped with the software package.

The neural network model consists of four convolutional layers with the number of output filters equal to 16, 32, 64, and 128, respectively, and two subsequent dense layers with sizes of 64 neurons each. Nonlinear activation functions for dense layers are set to rectified linear unit (ReLU). Each convolutional layer had a 0.2 dropout, valid pooling block, and steps per pooling equal to 1. The Adam optimizer was applied. Mean squared error was used as a loss function and pixel error as a metric. The custom pixel error function is , where *i* = 0,1 which corresponds to x and y axes, *j* is an index of an image in batch, *h* is the image size, and *T*_*ij*_ and *P*_*ij*_ are the true and predicted coordinates of the particle, correspondingly. The following parameters are used within the DeepTrack software to generate test images: Scatterer: PointParticle, Optics: Fluorescent microscope (NA = 0.7, wavelength = 660 nm, resolution = 1 × 10^–6^, magnification = 25, refractive index medium = 1.33, upscale = 2, padding = 30).

Since the size of test images is larger than the size used in the examples (100 × 100 versus 64 × 64), one more convolutional layer was added and the number of neurons in dense layers was doubled. Neural networks were trained on a data set of 20 000 images verified at 512 validation images. The batch size was equal to 64. The number of training epochs was set to 250 epochs with early stopping equal to 20 epochs. By setting an early stopping parameter, Keras was able to stop training in case of a stop of the loss function decrease. In reality, the number of training epochs did not exceed one hundred.

Background and random Poisson noise were added to bring images closer to the real experimental data (background = 1, Poisson noise min/max SNR 2/80). All generated images were augmented and had the size of 100 × 100 pixels. The output of the network is two parameters: *x* and *y* coordinates of the particle center.

### Feedback

The mechanical stabilization in brightfield microscopy was implemented on an existing ultrafast force-clamp system. The setup includes a custom-built microscope with a piezo-controlled stage (Physik Instrumente, P-527.2CL) and PIFOC objective scanner (Physik Instrumente, P-725.4CL). To reduce ambient noises, the system is built on a table resting on supports with pneumatic noise suppressors. Additionally, the microscope is placed on top of elastomeric dampers. The particles were imaged with water immersion objective (Nikon Plan-Apo 60×, N.A. 1.20) on a USB CMOS camera (Thorlabs DCC1545M). The pixel-to-nm ratio of the camera was 18.3 nm/pxl. *z*-Value calibration was obtained prior to the feedback test on the same particle by scanning the objective within a ±500 nm range (100 nm step size) followed by a linear fit of the calculated *z*-values.

Three-dimensional STORM imaging was performed on an inverted wide-field fluorescence microscope (Nikon ECLIPSE TE300) with 643 and 488 nm excitation lasers. Excitation was performed with inclined illumination through a TIRF 60× objective (Nikon 60×, oil immersion, NA 1.49 TIRF) to optimize the image contrast. Emitted fluorescence was collected through the same objective and imaged on an EMCCD camera (Andor iXon X3) after an additional 3× magnification. The full field of view is 40 × 40 μm^2^ wide, with an 80 nm pixel size.

### Fluorescent Microsphere Sample Preparation

Dragon Green beads (Bangslab, FSDG003, 0.51 m) were diluted at 0.1% (v/v) in 25 mM MOPS, 25 mM KCl, 4 mM MgCl_2_, 1 mM EGTA, and 1 mM DTT, pH 7.2. A chamber, composed by a standard glass coverslip sandwiched on a microscope slide with double-sided sticky tape, was filled with the bead solution and incubated for 5 min at room temperature. After a careful wash to remove unattached beads, the chamber was sealed with silicon grease and put on the microscope stage for measurements.

### STORM Imaging

The STORM sample consists of fixed HEK 293T cells with Dragon Green microspheres (Bangslab, FSDG003, 0.51 μm) as reference beads for the feedback algorithm. Cells were plated on poly-l-lysine-coated 18 mm diameter glass coverslips. After 24 h of incubation at 37 °C cells were fixed, by incubating with a 4% paraformaldehyde (PFA) solution for 10 min, permeabilized in 0.075% Triton X-100 solution for 7 min, and blocked with 4% bovine serum albumin (BSA) solution in PBS with added Ca^2+^ and Mg^2+^ for 30 min. After blocking, a dilution of Dragon Green beads at 0.1% (v/v) in 25 mM MOPS, 25 mM KCl, 4 mM MgCl_2_, 1 mM EGTA, and 1 mM DTT, pH 7.2, was incubated with cells on the coverslip for 2 min. Then the actin cytoskeleton was labeled by incubating overnight with 0.5 μM Alexa Fluor 647 phalloidin at 4 °C. After 20 h, the sample was washed once with PBS and mounted on an imaging chamber with imaging buffer composed of 200 mM β-mercaptoethylamine hydrochloride (MEA), 20% (v/v) sodium dl-lactate solution, and 3% (v/v) OxyFluor, in PBS pH 8. Prior to final rendering of the superresolved images localizations with a lateral uncertainty greater than 150 nm were filtered out. Final images were visualized at 10× magnification (i.e., the image pixel size is 8 nm). Images acquired with 3D STORM were reconstructed with ImageJ plugin ThunderSTORM. The details of the reconstruction parameters can be found in the Supporting Information.

## Supporting Information Available

Particle localization: derivation of local gradient algorithms for brightfield, astigmatism-based, and darkfield microscopy with calibration curve examples; variation of axial calibration curves for different particles; influence of bits and pixels resolution on algorithm performance; ThunderSTORM parameters; demonstration of selective properties of local gradient algorithms (PDF)

## Notes

A preprint of this manuscript: A. V. Kashchuk; O. Perederiy; C. Caldini; L. Gardini; F. S. Pavone; A. M. Negriyko; M. Capitanio. Particle localization using local gradients and its application to nanometer stabilization of a microscope. 2021, 468294. bioRxiv. 10.1101/2021.11.11.468294 (accessed November 9, 2022).
