# Supplementary material for: Particle Localization Using Local Gradients and Its Application to Nanometer Stabilization of a Microscope
Source: ACS Nano. 2022 Nov 16;17(2):1344–54. doi: 10.1021/acsnano.2c09787 (PMC9878972; doi:10.1021/acsnano.2c09787)
Supplement: Supplementary file 1 — nn2c09787_si_001.pdf [file nn2c09787_si_001.pdf]

Particle localization using local gradients and its application to nanometer stabilization of a microscope

Anatolii V. Kashchuk,[*,†,‡] Oleksandr Perederiy,[¶] Chiara Caldini,[‡] Lucia Gardini,[‡,§] Francesco Saverio Pavone,[†,‡,§] Anatoliy M. Negriyko,[¶] and Marco Capitanio[†,‡]

†*Department of Physics and Astronomy, University of Florence, Via Sansone 1, Sesto Fiorentino, 50019, Italy*

‡*LENS, European Laboratory for Non-Linear Spectroscopy, Via Nello Carrara 1, Sesto Fiorentino, 50019, Italy*

¶*Institute of Physics NASU, 46 Nauki Avenue, Kyiv, 03680, Ukraine*

§*National Institute of Optics—National Research Council, Largo Fermi 6, 50125, Florence, Italy*

E-mail: kashchuk@lens.unifi.it

Supplementary

Local gradient algorithm

For a given image $I(x, y)$, we define a local gradient of a pixel (x_i, y_j) as a centroid of all pixels within a circle of radius r $\{r \in \mathbb{R} \mid r > 0.5\}$ centered at the (x_i, y_j):

$$\left(g_x^{(i,j)}, g_y^{(i,j)}\right) = \left(\frac{\sum_{k,l=-r}^{r} I\left(x_{i+k}, y_{j+l}\right) x_{i+k}}{\sum_{k,l=-r}^{r} I\left(x_{i+k}, y_{j+l}\right)}, \frac{\sum_{k,l=-r}^{r} I\left(x_{i+k}, y_{j+l}\right) y_{j+l}}{\sum_{k,l=-r}^{r} I\left(x_{i+k}, y_{j+l}\right)} \right) \tag{S1}$$

By calculating gradients at each pixel the final gradient matrices are (also see Fig. 1):

$$G_{x,y} = \begin{pmatrix} g_{x,y}^{1,1} & g_{x,y}^{1,2} & \dots & g_{x,y}^{1,n} \\ \vdots & \vdots & \ddots & \vdots \\ g_{x,y}^{m,1} & g_{x,y}^{m,2} & \dots & g_{x,y}^{m,n} \end{pmatrix} \tag{S2}$$

Matrices G_x and G_y are x- and y-gradients correspondingly.

In most cases the calculation speed of the gradient can be increased by presenting the equations S1 and S2 as convolutions and applying the convolution theorem:

$$G_x = (I * X) \oslash S = \mathcal{F}^{-1}\left\{\mathcal{F}\{I\} \circ \mathcal{F}\{X\}\right\} \oslash S \tag{S3}$$

$$G_y = (I * Y) \oslash S = \mathcal{F}^{-1}\left\{\mathcal{F}\{I\} \circ \mathcal{F}\{Y\}\right\} \oslash S \tag{S4}$$

where "$\circ$" denotes Hadamard (element-wise) multiplication, "$\oslash$" denotes Hadamard division, $\mathcal{F}/\mathcal{F}^{-1}$ is a Fourier/inverse Fourier transform, "$*$" denotes convolution, X and Y are square matrices of size $k = 2\lceil r - 0.5 \rceil + 1$:

$$(X, Y) = \left(\begin{pmatrix} -r & -r+1 & \dots & r \\ -r & -r+1 & \dots & r \\ \vdots & \vdots & \ddots & \vdots \\ -r & -r+1 & \dots & r \end{pmatrix} \circ R, \begin{pmatrix} -r & -r & \dots & -r \\ -r+1 & -r+1 & \dots & -r+1 \\ \vdots & \vdots & \ddots & \vdots \\ r & r & \dots & r \end{pmatrix} \circ R \right) \tag{S5}$$

here R is a circular amplitude mask of the same size as X and Y. S is the sum of all

elements in each sub-image:

$$S = I(x,y) * J = \mathcal{F}^{-1}\{\mathcal{F}\{I\} \circ F\{J\}\} \tag{S6}$$

here J is a square matrix of ones of size k.

For most tracking applications the size of the window will remain constant and therefore, $\mathcal{F}\{X\}$, $\mathcal{F}\{Y\}$ and $\mathcal{F}\{J\}$ can be precalculated and reused to reduce the total calculation time.

x-y position detection

The x-y position of the particle is determined as an intersection of all gradient vectors in a least-square sense. A system of linear equations to find the least-square intersection of gradient lines $\boldsymbol{p}$ is:

$$\boldsymbol{L}\boldsymbol{p} = \boldsymbol{q} \tag{S7}$$

$$\boldsymbol{L} = \sum_{j=1}^{K} w_j \left(\boldsymbol{I} - \boldsymbol{n}_j \boldsymbol{n}_j^T\right), \boldsymbol{q} = \sum_{j=1}^{K} w_j \left(\boldsymbol{I} - \boldsymbol{n}_j \boldsymbol{n}_j^T\right) \boldsymbol{a}_j \tag{S8}$$

where w is a weighing vector, $\boldsymbol{I}$ is an identity matrix, $\boldsymbol{n}$ is a gradient direction vector, $\boldsymbol{a}$ is a coordinate vector of a point on the gradient line, K is a number of lines/equations. The solution of the system of linear equations (S7) returns the center of the particle.

z-calibration curve

Figure S1 shows a calibration curve for $3\mu m$ particle.

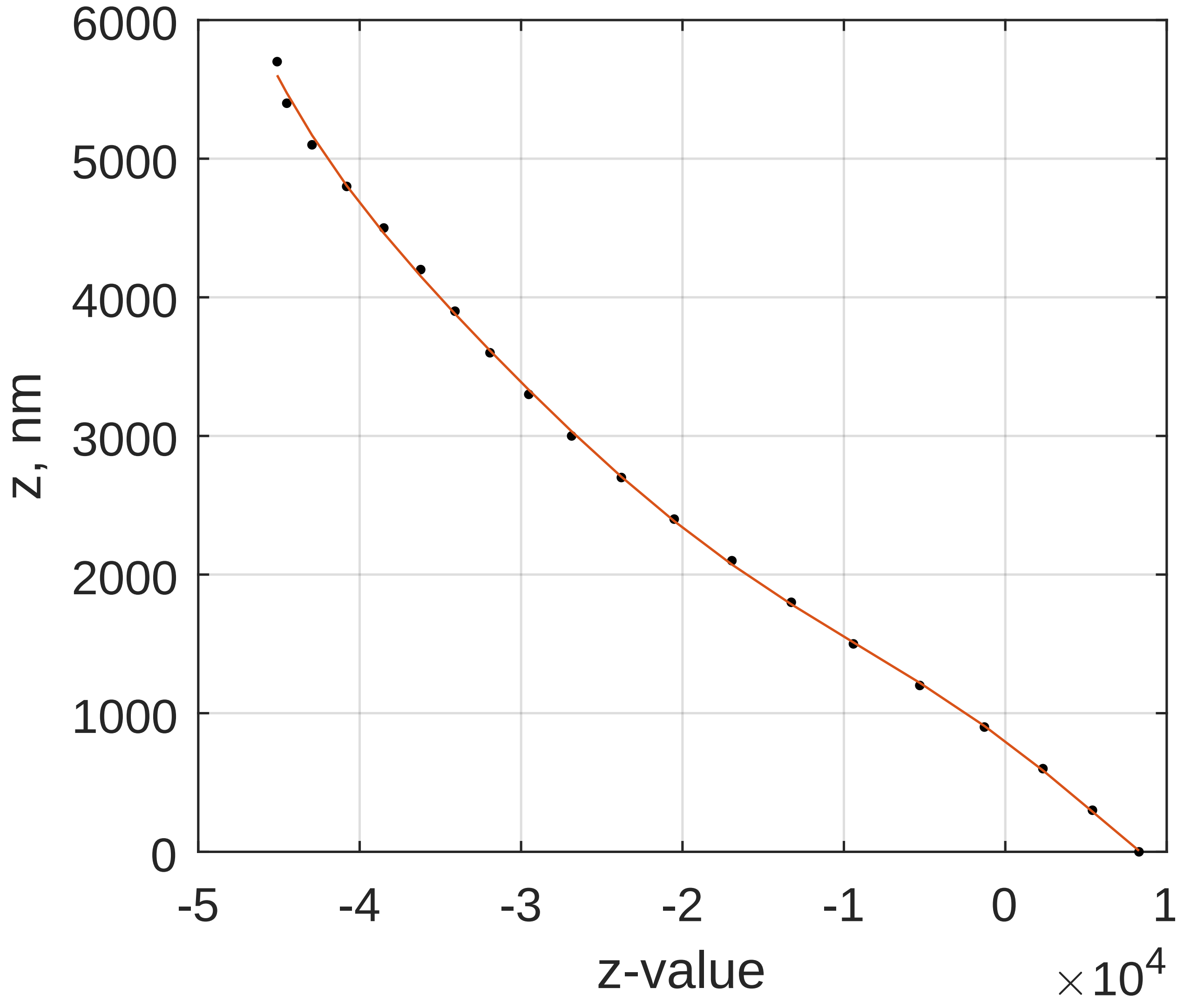

Figure S1: z-calibration curve for a polystyrene $3\mu m$ particle

z position detection in astigmatism based microscopy

Firstly, the x and y positions of the particle are calculated according to the previous section using the equations S3, S4 and S7. Then, a thresholded magnitude of local gradients is splitted vertically and horizontally relative to the calculated center. The gradient lines within each half are used in equation S7 to locate the position they are pointing to in a least-square sense. This results in four points which can be connected in two crossing axes similar to the minor and major axes in ellipse (however, in our case the axes may not be orthogonal). z-value is set as the length of the major axis with the sign determined as:

$$\begin{aligned} &\operatorname{sgn}(\sin(2\phi)(x_1 - x_3) + \cos(2\phi)(y_1 - y_3)) - \\ &\qquad - \cos(2(\phi + \pi/2))(x_4 - x_2) + \sin(2(\phi + \pi/2))(y_4 - y_2)) \end{aligned} \quad \text{(S9)}$$

where ϕ is the angle of the major axis which corresponds to positive z-values, $x_1 ... x_4$, $y_1 ... y_4$ are coordinates of the calculated centers for each half.

z position detection in darkfield microscopy

Figure S2 shows an axial calibration curve of a gold nanoparticle in a dark field in astigmatism-based microscopy. Similarly to other tests, the particle images were recorded at different axial positions with step of $10nm$. The calibration was performed on each second image (i.e. with a step size of $20nm$) and the other images are used for validation and error calculation.

Gold nanoparticles are spheres (Sigma-Aldrich 742015) with core size $57nm - 63nm$ (mean diameter $60nm$)

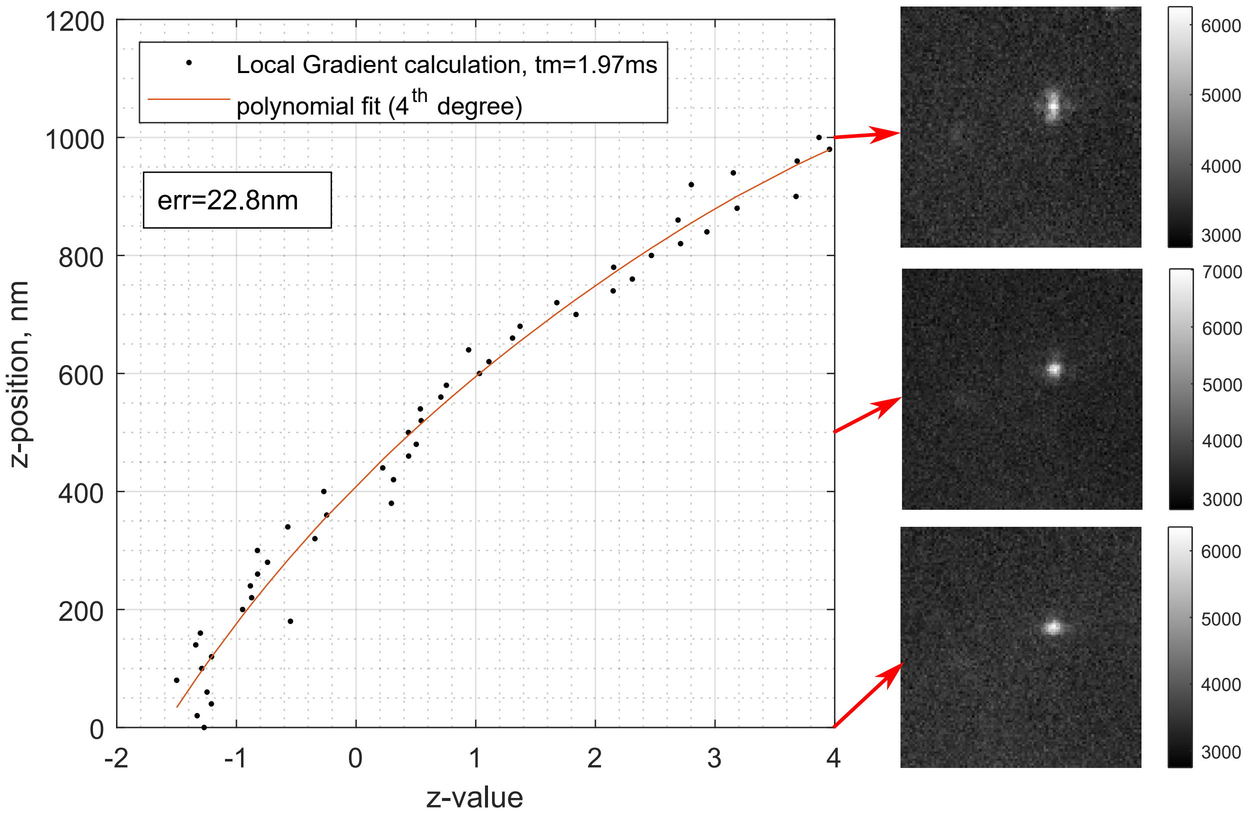

Figure S2: z-calibration curve for a gold nanoparticle of 60nm diameter. Insets show images of particle at different axial positions: 0, 500 and 1000 nanometers. tm shows an average time for algorithm to execute in Matlab environment. The size of the analyzed images is $100 \times 100 pxls$

Axial calibration variation

Figure S3 demonstrates a variation in the axial calibration curves of different particles recorded at the same time. A 60nm gold nanoparticles are imaged in a darkfield illumination in astigmatism-based microscopy with an axial step size of 20nm. It is clear that even for the same sample the calibration curves have different biases but similar shapes and slopes. The mean standard error of the calibration curves of different particles is 43.8nm

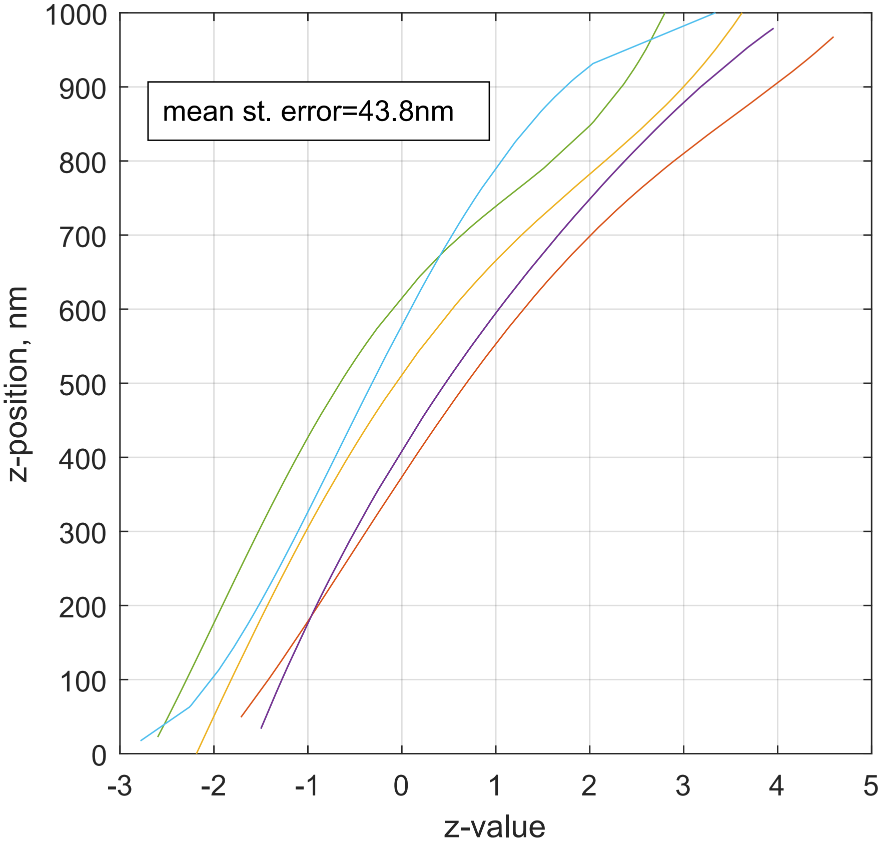

Figure S3: A set of six axial calibration curves measured on a different gold nanoparticles in darkfield illumination. Each particle is measured at the same time at different locations across the field of view. mean st. error shows a standard error averaged across all z-positions

Bits and pixels resolution

Figure S4 demonstrates the performance of the LoG algorithm on simulated images at varying bit resolutions of the image for different pixel resolutions. The particle is modeled as a Gaussian function with corresponding standard deviations of 1, 2.5 and 5 pixels randomly placed on the image with added noise of specified SNR. A set of 20 images (100×100pxls) was generated for each SNR (12 SNR levels). The error is defined as a distance between the predicted and true location of the particle. The signal level in the SNR is defined as the maximum intensity for each generated image. As can be expected, the accuracy of the localization decreases as the size resolution decreases (i.e. particle is represented by fewer pixels). The bit resolution has no apparent effect on the algorithm performance.

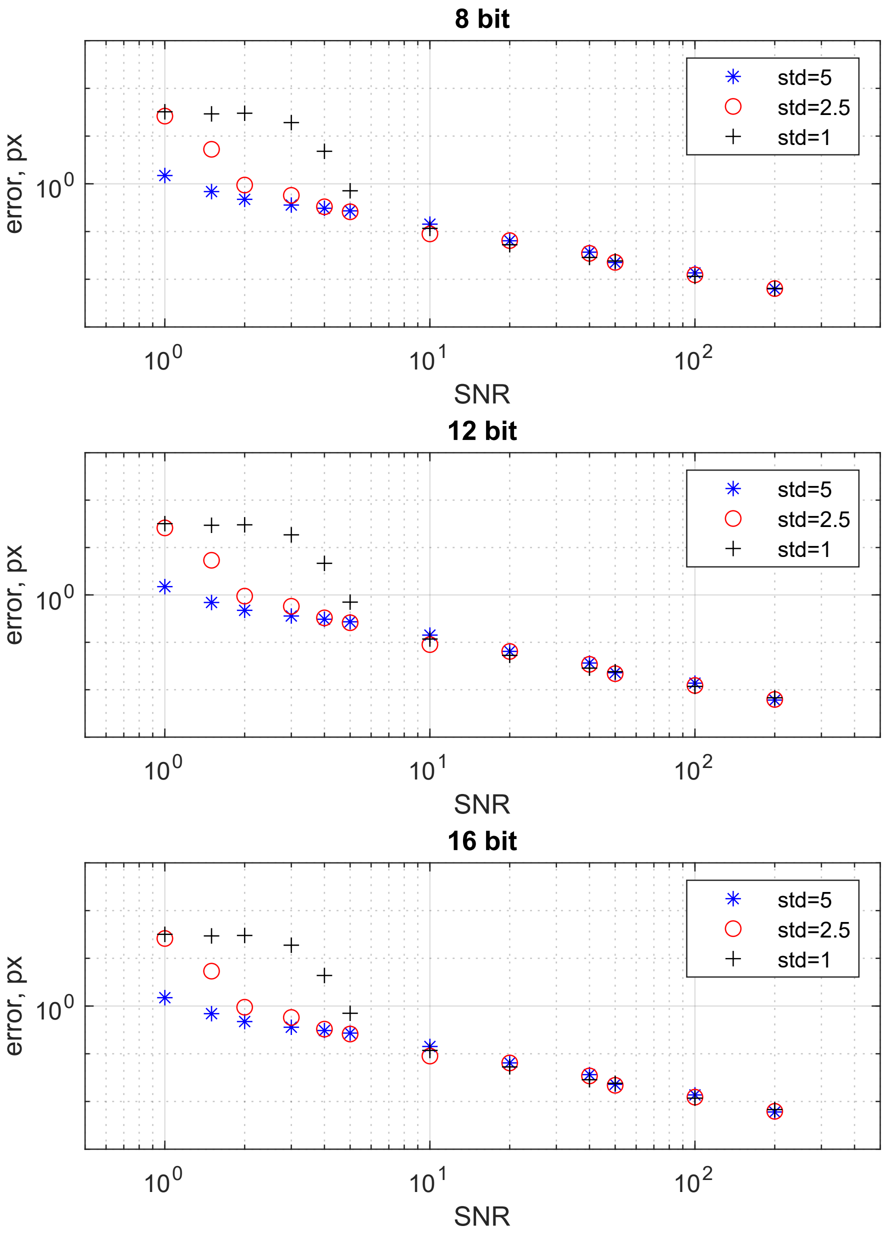

Figure S4: Algorithm performance on generated images with added noise at different bit resolutions (8,12 and 16 bits) and pixel resolutions (standard deviation of the Gaussian 1, 2.5, 5 pxls)

Thunderstorm parameters for analysis and visualization

Images acquired with 3D STORM were reconstructed through ImageJ plugin ThunderSTORM using the following parameters:

Prior to final rendering of the super-resolved images localizations with a lateral uncertainty greater than 150 nm were filtered out. Final images were visualized at 10x magnifi-

Table 1: Thunderstorm parameters

Image filtering	**Filter**: Gaussian filter<br>**Sigma** [px]: 3.0
Approximate localization of molecules	**Method**: Local maximum<br>**Peak intensity threshold**: 6.5std(Wave.F1)<br>**Connectivity**: 8-neighbourhood
Sub-pixel localization of molecules	**Method**: PSF: Elliptical Gaussian (3D astigmatism)<br>**Fitting radius** [px]: 11<br>**Fitting method**: Maximum likelihood<br>**Initial sigma** [px]: 3.0
Visualization of the results	**Method**: Normalized Gaussian<br>**Magnification**: 10<br>**Update frequency** [frames]: 50<br>**3D**<br>**Colorize z-stack**<br>**Z range** (from:step:to) [nm]: -1000:10:1000<br>**Lateral uncertainty** [nm]: 10<br>**Axial uncertainty** [nm]: 20

cation (i.e. the image pixel size is 8 nm).

Selective properties of local gradient algorithm

The Figure S5 shows the selective property of the local gradient algorithms. By varying the window size r one can control the enhancement in the gradient of the corresponding particle.

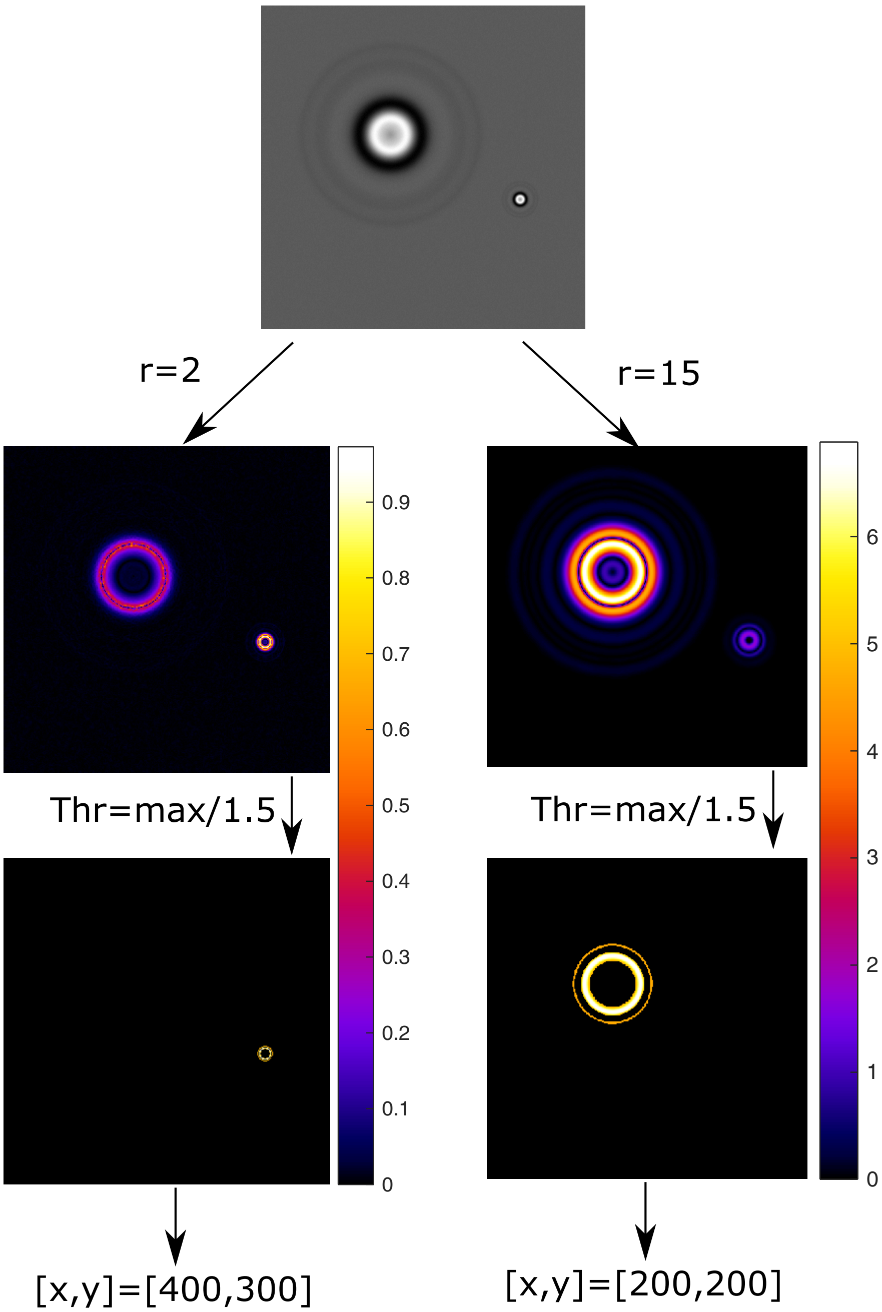

Figure S5: Size selectivity of local gradient algorithm. Two particles of different sizes are present in the field of view. Two LoG results with $r = 2, threshold = max/1.5$ and $r = 15, threshold = max/1.5$ (max is the maximum gradient for current image) that enhance smaller and bigger particles are shown. In both cases the thresholding of the gradients leads to the correct location estimation for each particle correspondingly.
